# A Tutorial on Analysis and Simulation of Boolean Gene Regulatory Network Models

**DOI:** 10.2174/138920209789208237

**Published:** 2009-11

**Authors:** Yufei Xiao

**Affiliations:** 1 Dept. of Epidemiology & Biostatistics, University of Texas Health Science Center at San Antonio, San Antonio, TX 78229, USA; 2 Greehey Children's Cancer Research Institute, University of Texas Health Science Center at San Antonio, San Antonio, TX 78229, USA

## Abstract

Driven by the desire to understand genomic functions through the interactions among genes and gene products, the research in gene regulatory networks has become a heated area in genomic signal processing. Among the most studied mathematical models are Boolean networks and probabilistic Boolean networks, which are rule-based dynamic systems. This tutorial provides an introduction to the essential concepts of these two Boolean models, and presents the up-to-date analysis and simulation methods developed for them. In the Analysis section, we will show that Boolean models are Markov chains, based on which we present a Markovian steady-state analysis on attractors, and also reveal the relationship between probabilistic Boolean networks and dynamic Bayesian networks (another popular genetic network model), again *via *Markov analysis; we dedicate the last subsection to structural analysis, which opens a door to other topics such as network control. The Simulation section will start from the basic tasks of creating state transition diagrams and finding attractors, proceed to the simulation of network dynamics and obtaining the steady-state distributions, and finally come to an algorithm of generating artificial Boolean networks with prescribed attractors. The contents are arranged in a roughly logical order, such that the Markov chain analysis lays the basis for the most part of Analysis section, and also prepares the readers to the topics in Simulation section.

## 1. INTRODUCTION

In most living organisms, genome carries the hereditary information that governs their life, death, and reproduction. Central to genomic functions are the coordinated interactions between genes (both the protein-coding DNA sequences and regulatory non-coding DNA sequences), RNAs and proteins, forming the so called gene regulatory networks (or genetic regulatory networks).

The urgency of understanding gene regulations from systems level has increased tremendously ever since the early stage of genomics research. A driving force is that, if we can build good gene regulatory network models and apply intervention techniques to control the genes, we may find better treatment for diseases resulting from aberrant gene regulations, such as cancer. In the past decade, the invention of high throughput technologies has made it possible to harvest large quantities of data efficiently, which is turning the quantitative study of gene regulatory networks into a reality. Such study requires the application of signal processing techniques and fast computing algorithms to process the data and interpret the results. These needs in turn have fueled the development of genomic signal processing and the use of mathematical models to describe the complex interactions between genes.

The roles of mathematical models for gene regulatory networks include:

- Describing genetic regulations at a system level;
- Enabling artificial simulation of network behavior;
- Predicting new structures and relationships;
- Making it possible to analyze or intervene in the network through signal processing methods.

Among various mathematical endeavors are two Boolean models, Boolean networks (BNs) [1] and probabilistic Boolean networks (PBNs) [2], in which each node (gene) takes on two possible values, ON or OFF (or 1 and 0), and the way genes interact with each other is formulated by standard logic functions. They constitute an important class of models for gene regulatory networks, in that they capture some fundamental characteristics of gene regulations, are conceptually simple, and their rule-based structures bear physical and biological meanings. Moreover, Boolean models can be physically implemented by electronic circuits, and demonstrate rich dynamics that can be studied using mathematical and signal processing theory (for instance, Markov chains [2, 3]).

In practice, Boolean models have been successfully applied to describe real gene regulatory relations (for instance, the *drosophila* segment polarity network [4]), and the attractors of BNs and PBNs have been associated with cellular phenotypes in the living organisms [5]. The association of network attractors and actual phenotypes has inspired the development of control strategy [6] to increase the possibility of reaching desirable attractors (“good” phenotypes) and decrease the likelihood of undesirable attractors (“bad” phenotypes such as cancer). The effort of applying control theory to Boolean models is especially appealing in the medical community, as it holds potential to guide the effective intervention and treatment in cancer.

The author would like to bring the fundamentals of Boolean models to a wider audience in light of their theoretical value and pragmatic utility. This tutorial will introduce the basic concepts of Boolean networks and probabilistic Boolean networks, present the mathematical essentials, and discuss some analyses developed for the models and the common simulation issues. It is written for researchers in the genomic signal processing area, as well as researchers with general mathematics, statistics, engineering, or computer science backgrounds who are interested in this topic. It intends to provide a quick reference to the fundamentals of Boolean models, allowing the readers to apply those techniques to their own studies. Formal definitions and mathematical foundations will be laid out concisely, with some in-depth mathematical details left to the references.

## 2. PRELIMINARIES

In Boolean models, each variable (known as a *node*) can take two possible values, 1 (ON) and 0 (OFF). A node can represent a gene, RNA sequence, or protein, and its value (1 or 0) indicates its measured abundance (expressed or unexpressed; high or low). In this paper, we use “node” and “gene” interchangeably.

A *state* in Boolean models is a binary vector of all the gene values measured at the same time, and is also called the gene activity (or expression) profile (GAP). The *state space* of a Boolean model consists of all the possible states, and its size will be 2*^n^* for a model with *n* nodes.

**Definition 1**[2, 7] A Boolean network is defined on a set of *n* binary-valued nodes (genes) $V=\left\{x_{1},\cdot \cdot \cdot ,x_{n}\right\},x_{i}\in \left\{0,1\right\}$, where each node *x_i_* has *k_i_* parent nodes (regulators) chosen from *V*, and its value at time *t* + 1 is determined by its parent nodes at *t* through a Boolean function *f_i_*,

(1) $$x_{i}\left(t+1\right)=f_{i}\left(x_{\mathrm{i1}}\left(t\right),x_{\mathrm{i2}}\left(t\right),...,x_{{\mathrm{ik}}_{i}}\left(t\right)\right),\left\{i1,\cdot \cdot \cdot ,{\mathrm{ik}}_{i}\right\}⫅\left\{1,\cdot \cdot \cdot ,n\right\}$$

*k_i_* is called the *connectivity* of *x_i_*, and *f_i_* is the regulatory function. Defining network function **f** = (*f*_1_,...,*f_n_*), we denote the Boolean network as *β*(*V*, **f**). Let the network state at time *t* be **x**(*t*) = (*x*_1_(*t*),...,*x_n_*(*t*)), the state transition **x**(*t*) → **x**(*t* + 1) is governed by **f**, written as **x**(*t* + 1) = **f**(**x**(*t*)).

In Boolean networks, genetic interactions and regulations are hard-wired with the assumption of biological determinism. However, any gene regulatory network is not a closed system and has interactions with its environment and other genetic networks, and it is also likely that genetic regulations are inherently stochastic; therefore, Boolean networks will have limitations in their modeling power. Probabilistic Boolean networks were introduced to address this issue [2, 7], such that they are composed of a family of Boolean networks, each of which is considered a context [8]. At any given time, gene regulations are governed by one component Boolean network, and network switchings are possible such that at a later time instant, genes can interact under a different context. In this sense, probabilistic Boolean networks are more flexible in modeling and interpreting biological data.

**Definition 2** [2, 3, 7] A probabilistic Boolean network is defined on $V=\left\{x_{1},\cdot \cdot \cdot ,x_{n}\right\},x_{i}\in \left\{0,1\right\}$ , and consists of *r* Boolean networks *β*_1_(*V*,**f**_1_),...,*β_r_*(*V*,**f***_r_*), with associated network selection probabilities *c*_1_,...,*c_r_* such that $\sum_{j=1}^{r}c_{j}=1$. The network function of the *j* -th BN is ${\text{f}}_{j}=\left(f_{j}^{\left(1\right)},...,f_{j}^{\left(n\right)}\right)$ . At any time, genes are regulated by one of the BNs, and at the next time instant, there is a probability *q* (switching probability) to change network; once a change is decided upon, we choose a BN randomly (from *r* BNs) by the selection probabilities. Let *p* be the rate of random gene perturbation (flipping a gene value from 0 to 1 or 1 to 0), the state transition of PBN at *t* (assuming operation under *β_j_*) is probabilistic, namely [3],

(2) $$\text{x}\left(t+1\right)=\left\{\begin{array}{c} {\text{f}}_{j}\left(\text{x}\left(t\right)\right),\;\mathrm{with probability}\;{\left(1-p\right)}^{n},\\ \text{x}\left(t\right)⊕\gamma ,\;\mathrm{with probability}\;1-{\left(1-p\right)}^{n}, \end{array}\right.$$

where ⊕ is bit-wise modulo-2 addition, *γ*=(*γ*_1_,...,*γ_n_*) is a random vector with P*r*{*γ_i_*=1}= *p*, and **x**(*t*)⊕*γ* denotes a random perturbation on the state ***x***(*t*) (one or more genes are flipped). Let the set of network functions be **F**={**f**_1_,...,**f***_r_*}, and we denote the PBN by *G*(*V*,**F**,**c**,*p*) (see Remark 1).

Alternatively, the PBN can be represented as *G*(*V*,Ψ,*α*,*p*), with Ψ = {Ψ_1_,...,Ψ_n_} and *α*={*α*_1_,...,*α_n_*}. In this representation, each node *x_i_* is regarded as being regulated by a set of *l*(*i*) Boolean functions $\psi_{i}=\left\{\psi_{1}^{\left(i\right)},\cdot \cdot \cdot \psi_{l\left(i\right)}^{\left(i\right)}\right\}$ with the corresponding set of function selection probabilities $\alpha_{i}=\left\{\alpha_{1}^{\left(i\right)},...,\alpha_{l\left(i\right)}^{\left(i\right)}\right\}\left(\sum_{j=1}^{l\left(i\right)}\alpha_{j}^{\left(i\right)}=1\right)$ . The two representations are related such that any network function **f***_j_* is a realization of the regulatory functions of *n* genes by choosing one function from the function set Ψ*_i_* for each gene *x_i_*, and we can write

(3) $${\text{f}}_{j}=\left(\psi_{j_{1}}^{\left(1\right)},...,\psi_{j_{n}}^{\left(n\right)}\right),j_{i}\in \left\{1,...,l\left(i\right)\right\}.$$

Moreover, if it is an independent PBN, namely $\mathrm{Pr}\left\{\psi_{j_{1}}^{\left(1\right)},...,\psi_{j_{n}}^{\left(n\right)}\right\}=\prod_{i=1}^{n}\mathrm{Pr}\left\{\psi_{j_{i}}^{\left(i\right)}\right\}$ , **c** and *α* are related by

(4) $$c_{j}=\prod_{i=1}^{n}\alpha_{j_{i}}^{\left(i\right)}.$$

**Remark 1 ***q* does not appear in the PBN representation, because according to the network switching scheme described, it can be shown that the probability of being in the *β_j_* at any time is equal to *c_j_*, regardless of *q*. However, if we modify the network switching scheme such that, once a network switch is decided upon, we randomly choose any network *other than* the current network, it will require the definition of *r*(*r*–1) conditional selection probabilities, $c_{\mathrm{jk}}=\mathrm{Pr}\left\{{\text{f}}_{k}\left|{\text{f}}_{j}\right.\right\},k,j\in \left\{1,...,r\right\},k\neq j$ , and the derivation of Pr{**f_j_**} (the probability of being in *β_j_*) is left as an exercise to the reader.

A Boolean model with finite number of nodes has a finite state space. From the definition of Boolean network, it follows that its state transitions are deterministic, that is, given a state, its successor state is unique. Naturally, if we represent the whole state space and the transitions among the sates of a BN graphically, we can have a *state transition diagram*.

**Definition 3 **The state transition diagram of an *n*-node Boolean network *β*(*V*, **f**) is a directed graph *D*(*S*,*E*). *S* is a set of 2*^n^* vertices, each representing a possible state of a Boolean network; *E* is a set of 2^n^ edges, each pointing from a state to its successor state in state transition. If a state transits to itself, then the edge is a loop. The state transitions are computed by evaluating **x**(*t*+1) = **f**(**x**(*t*)) exactly 2*^n^* times, each time **x**(*t*) being 00...0,00...1,...,11...1 respectively.

Fig. (**1**) is an example of state transition diagram of a three-node BN. Like BNs, a PBN also has finite state space. Although state transitions in a PBN are not deterministic, they can be represented probabilistically. We will show how to construct the state transition diagram of a PBN in the Simulation section.

With the help of state transition diagram, such as the one in Fig. (**1**), we can easily visualize that in a BN, any state trajectory in time **x**(0)→ **x**(1)→ **x**(2)→ ... must end up in a “trap”, and stay there forever unless a gene perturbation occurs. Similarly, if neither gene perturbation nor network switching has occurred, a time trajectory in a PBN will end up in a “trap” in one of the component BNs too; however, either gene perturbation or a network switch may cause it to escape from the trap. In spite of this, when gene perturbation and network switching are rare, a PBN is most likely to reach a “trap” before either occurs and will spend a reasonably long time there.

**Definition 4 **Starting from any initial state in a finite Boolean network, when free of gene perturbation, state transitions will allow the network to reach a finite set of states {**a**_1_,...,**a**_*m*_} and cycle among them in a fixed order forever. The set of states is called an attractor, denoted by *A*. If *A* contains merely one state, it is a singleton attractor; otherwise, it is an attractor cycle. The set of states from which the network will eventually reach an attractor *A* constitutes the basin of attraction of *A*. A BN may have more than one attractor.

The attractors of a PBN are defined as the union of attractors of its component BNs. In particular, if a PBN is composed of *r* BNs, and the *k* -th BN has *m_k_* attractors, *A_k1_* ,*A_k2_*,..., *A_km_k__* , then the attractors of PBN are $\left\{A_{11},A_{12},...,A_{{1m}_{1}}\right\}\cup \cdot \cdot \cdot \cup \left\{A_{\mathrm{r1}},A_{\mathrm{r2}},...,A_{{\mathrm{rm}}_{r}}\right\}$ .

In a BN, different basins of attraction are depicted in the state transition diagram as disjoint subgraphs. In Fig. (**1**), *D*(*S*, *E*) is composed of three disjoint subgraphs, *D*_1_(*S*_1_,*E*_1_), *D*_2_(*S*_2_,*E*_2_) , and *D*_3_(*S*_3_,*E*_3_). 110 and 101 are singleton attractors, while 100 and 111 constitute a cycle. Their respective basins of attraction are *S*_1_ = {000,010,110}, *S*_2_ = {101} and *S*_3_ = {001,011,100,111}.

We are interested in the attractors of a Boolean model for at least two reasons: (1) Attractors represent the stable states of a dynamic system, thus they are tied to the long term behavior of Boolean models; (2) Earlier researchers demonstrated the association of cellular phenotype with attractors [5], thus giving a biological meaning to the attractors. Intuitively, when an attractor has a large basin of attraction, the corresponding phenotype is more likely than that of an attractor with much smaller basin of attraction. To develop intervention strategies that change the long term behavior of Boolean models, it is important to study the attractors.

## 3. ANALYSES OF BOOLEAN MODELS

Although analysis and simulation are two parallel subjects with Boolean models, the former includes some essential results that lay a foundation for the latter. In this section, we visit Boolean model analysis first.

One of the central ideas with Boolean models is their connection with Markov chains (subsection 3.1). Because of this, Boolean models, under certain conditions, possess steady-state distributions. The steady-state probabilities of attractors, which indicate the long-run trend of network dynamics, can be found analytically *via *Markov chain analysis (subsection 3.2). Moreover, the relationship between PBNs and Bayesian networks (another class of gene regulatory network models) can be established in a similar manner (subsection 3.3). Lastly, a subsection will be dedicated to structural analysis, which opens a door to other topics beyond this tutorial (such as control of genetic networks).

### 3.1. Markov Chain Analysis

As readers will find out soon, the transition probability matrix introduced below is not only a convenience in Markov chain analysis, but also finds itself useful in simulation, to be discussed in Section 4.

#### 3.1.1. Transition Probability Matrix

On a Boolean model of *n* nodes, a *transition probability matrix* $T={\left[t_{\mathrm{ij}}\right]}_{2^{n}\times 2^{n}}$ can be defined where *t_ij_* indicates the probability of transition from one state (which is equal to *i*–1 if we convert the binary vector to an integer) to another state (which corresponds to *j*–1).

In a Boolean network *β*(*V*,**f**), *t_ij_* can be computed by

(5) $$t_{\mathrm{ij}}=\left\{\begin{array}{c} 1,\exists s\in {\left\{0,1\right\}}^{n}\text{such that dec}\left(s\right)=i-1,\mathrm{dec}\left(f\left(s\right)\right)=j-1,\\ 0,\text{otherwise}, \end{array}\right.$$

where dec(.) converts a binary vector to an integer, for instance, dec(00101) = 5. Since BN is deterministic, *T* contains one 1 on each row, and all other elements are 0's.

In a PBN consisting of *r* BNs *β*_1_(*V*,**f**_1_),...,*β_r_*(*V*,**f***_r_*), *t_ij_* can be computed as follows [2, 3]. Note that *p* (random gene perturbation rate) and *γ* are defined as in Definition 2, and *c_k_* is the selection probability of *β_k_*.

$t_{\mathrm{ij}}=\sum_{k=1}^{r}\mathrm{Pr}\left\{\beta_{k}\mathrm{is selected}\right\}.$

(6) $$\begin{array}{c} \mathrm{Pr}\left\{\text{s}\to \text{w},\mathrm{dec}\left(\text{s}\right)=i-1,\mathrm{dec}\left(\text{w}\right)=j-1\left|\beta_{k}\text{ is selected }\right.\right\}\\ =\sum_{k=1}^{r}c_{k}.\left[\mathrm{Pr}\left\{\text{s}\to w\text{ by state transition },\mathrm{dec}\left(\text{s}\right)=i-1,\mathrm{dec}\left(\text{w}\right)=j-1\left|{\text{f}}_{k}\right.\right\}\right.\\ +\mathrm{Pr}\left.\left\{\text{s}\to w\text{ by random gene perturbation },\text{ dec }\left(\text{s}\right)=\text{i}-1,\mathrm{dec}\left(\text{w}\right)=j-1\left|{\text{f}}_{k}\right.\right\}\right]\\ =\sum_{k=1}^{r}c_{k}.\left[{\left(1-p\right)}^{n}\mathrm{Pr}\left\{\text{s}\to {\text{f}}_{k}\left(\text{s}\right),\mathrm{dec}\left(\text{s}\right)=i-1,\mathrm{dec}\left({\text{f}}_{k}\left(\text{s}\right)\right)=j-1\left|{\text{f}}_{k}\right.\right\}\right.\\ \left.+\mathrm{Pr}\left\{\text{s}\to \text{s}⊕\gamma ,\gamma \neq \left(0,...,0\right),\mathrm{dec}\left(\text{s}\right)=i-1,\mathrm{dec}\left(s⊕\gamma \right)=j-1\left|{\text{f}}_{k}\right.\right\}\right]\\ =\sum_{k=1}^{r}c_{k}.\left[{\left(1-p\right)}^{n}1_{\left\{\text{s}\to {\text{f}}_{k}\left(\text{s}\right),\mathrm{dec}\left(\text{s}\right)=i-1,\mathrm{dec}\left({\text{f}}_{k}\left(\text{s}\right)\right)=j-1\right\}}+p_{\gamma }1_{\left[i\neq j\right]}\right], \end{array}$$

where **1** 's are indicator functions, $p_{\gamma }=\left(\begin{array}{c} n\\ l \end{array}\right)p^{l}{\left(1-p\right)}^{n-1},$ , *l*=number of 1's in the random vector *γ*=(*γ*_1_,...,*γ_n_*), and *l* indicates the Hamming distance between **s** and **w**.

When taking a closer look at Eq. (6), we find that *T* is the sum of a fixed transition matrix $\overset{-}{T}$ and a perturbation matrix $\tilde{T}$ ,

(7) $$\bar{T}={\left(1-p\right)}^{n}\sum_{j=1}^{r}c_{j}T_{j},$$

(8) $$\tilde{T}=\left[{\tilde{t}}_{\mathrm{ij}}\right],{\tilde{t}}_{\mathrm{ij}}=n\left(\begin{array}{c} n\\ n_{\mathrm{ij}} \end{array}\right)p^{\eta_{\mathrm{ij}}}{\left(1-p\right)}^{{n-\eta }_{\mathrm{ij}}}1_{\left[i\neq j\right]},$$

where *T_j_* and *c_j_* are the transition probability matrix and the network selection probability of the *j* -th Boolean network, respectively; *η_ij_* is the Hamming distance between states **s** and **w**, with dec(**s**) = *i*–1 and dec(**w**) = *j*–1.

*T_j_* is sparse with only 2*^n^* non-zero entries (out of 2*^n^*×2*^n^* entries), where each is a state transition driven by the network function **f**_*j*_ and involves *n* computations. $\tilde{T}$ depends only on *n* and *p* , and involves *n* computations. Thus, the computational complexity for *T* is $O\left({n\cdot r\cdot 2}^{n}\right)$ [9].

#### 3.1.2. Boolean Models are Markov Chains

Given the definition of *T* matrix in Section 3.1.1, we can see that a Boolean model with *n* genes is a homogeneous Markov chain of *N* = 2*^n^* states, with *T* being the Markov matrix and $\sum_{j=1}^{2^{n}}t_{\mathrm{ij}}=1,\forall i$ . A state **x** (a binary vector of length *n*) in Boolean model has one-to-one correspondence with the *i* -th state (1 ≤ *i* ≤ *N*) in the associated Markov chain by dec(**x**) = *i*–1.

What is the use of matrix *T* ? Let *W* = *T^n^*, we can show that the (*i,j*) -th element of *W* is equal to the probability of transition from the *i* -th state to the *j* -th state of the Markov chain in *n* steps, $w_{\mathrm{ij}}=\mathrm{Pr}\left\{\text{x}\left(t+n\right)=\text{z},\mathrm{dec}\left(\text{z}\right)=j-1\left|\text{x}\left(t\right)=\text{y},\mathrm{dec}\left(\text{y}\right)=i-1\right.\right\}$ .

The proof is left as an exercise to the reader.

An *N* -state Markov chain possesses a *stationary distribution* (or invariant distribution) if there exists a probability distribution *π* = (*π*_1_,...,*π_N_*) such that

*π* = *πT*.

*π* = *πT* implies $\pi ={\pi T}^{n},\forall n$. Thus in a Markov chain with stationary distribution *π* , if we start from the i -th state with probability *π_i_*, the chance of being in any state *j* after an arbitrary number of steps is always *π_j_* .

An *N* -state Markov chain possesses a *steady-state distribution* $\pi^{*}=\left(\pi_{1}^{*},...,\pi_{N}^{*}\right)$ if starting from any initial distribution *π*

$\pi^{*}=\underset{k\to \infty }{\text{lim}}\pi T^{k},$

it means that regardless of the initial state, the probability of a Markov chain being in the *i* -th state in the long run is $\pi_{i}^{*}$ . A Markov chain possessing a stationary distribution does not necessarily possess a steady-state distribution.

Why should it be of our concern if the Markov chain has a steady-state distribution or not? This is because we are interested in the Boolean model associated with the Markov chain, and would like to know how it behaves in the long-run. As a reminder, the attractors of a Boolean model are often associated with cellular phenotype, and by finding out the steady-state probabilities of a given attractor, we can have a general picture of the likelihood of a certain phenotype. When a Boolean model possesses (namely, its Markov chain possesses) a steady-state distribution, we can find those probabilities by simulating the model for a long time, starting from an arbitrary initial state **x**(0). In fact, this implies the equivalence of “space average” and “time average”, as is a common concept in stochastic processes.

When will a Markov chain possess a steady-state distribution? It turns out that an *ergodic* Markov chain will do. A Markov chain is said to be ergodic if it is irreducible and aperiodic [10].

**Definition 5 **A Markov chain is irreducible if it is possible to go from every state to every state (not necessarily in one move). 

**Definition 6 **In a Markov chain, a state has period *d* if starting from this state, we can only return to it in *n* steps and *n* is a multiple of *d*. A state is periodic if it has some period > 1. A Markov chain is aperiodic if none of its state is periodic. 

A Boolean network possesses a stationary distribution, but not a steady-state distribution unless it has one singleton attractor and no other attractors. Here we show how to find a stationary distribution. Assume a BN has *m* singleton attractors, **a**_1_,...,**a***_m_*, or an attractor cycle {**a**_1_,...,**a***_m_*}, where dec(**a**_1_) = *i*_1_–1,...,dec(**a**_*m*_)=*i_m_*–1, then *π* with $\pi_{i_{1}}=...=\pi_{i_{m}}=1/m$ and $\pi_{j}=0,j\notin \left\{i_{1},...,i_{m}\right\}$ is a stationary distribution (the proof is left as an exercise to the reader). If a BN has a combination of singleton attractors and cycles, *π* can be constructed such that the probabilities corresponding to the singleton attractors are equal, the probabilities corresponding to the states within each attractor cycle are equal, and $\sum_{i=1}^{N}\pi_{i}=1$ . When there is only one attractor in the BN, the stationary distribution is unique.

When *p*,*q*> 0, a PBN possesses steady-state distribution, because the Markov chain corresponding to the PBN is ergodic. Interested readers can find the proof in [3]. Now that PBN has a steady-state distribution, we can obtain such distribution in two ways: (2) solving the linear equations $\pi \left(T-I\right)=0,\sum_{i=1}^{N}\pi_{i}=1$ (*I* is the identity matrix), and interested readers can consult books on linear algebra; (2) using the empirical methods in Section 4.3. If we are interested in the steady-state probabilities of the attractors only, an analytic method exists, to be discussed next.

### 3.2. Analytic Method for Computing the Steady-State Probabilities of Attractors

Recall from Section 2 that attractors are important to the long-term behavior of Boolean models because they are associated with cellular phenotypes; now we also know that PBNs possess steady-state distributions, which means that a PBN has a unique long-term trend independent of initial state. Therefore, we would naturally ask the question, how can we find the long-term probabilities of these attractors which are so important to us?

In the following, we will present a Markov chain based analytic method that answers this question, and more details, including proofs, can be found in [11].

#### 3.2.1. Steady-State Distributions of Attractors in a BN with Perturbations

First consider a special case of PBN, Boolean network with perturbations (BNp), in which any gene has a probability *p* of flipping its value. A BNp inherits all the attractors and corresponding basins of attraction from the original BN. Because of the random gene perturbations, BNps possess steady-state distributions (the proof is similar to that of PBN, and it is left as an exercise to the reader).

A BNp defined on *V* = {*x*_1_,...,*x_n_*} with gene perturbation rate *p* can be viewed as homogenous irreducible Markov chain **X_*t*_** with state space {0,1}*^n^*. Let $\text{x},\text{y}\in {\left\{0,1\right\}}^{n}$ be any two states, then at any time *t*,*P*_y_(**x**)=Pr{**X**_t+1_=**x**|**X***_t_*=**y**}  is the probability of state transition from **y** to **x**.

For **X***_t_*, there exists a unique steady-state distribution *π*. Let the steady-state probability of state **x** be *π*(**x**), and let $B\subset {\left(0,1\right)}^{n}$ be a collection of states, then the steady-state probability of *B* is $\pi \left(B\right)=\sum_{\text{x}\in B}\pi \left(\text{x}\right)$ .

Assume the BNp has attractors *A*_1_,...,*A_m_*, with corresponding basins of attraction (or simply referred to as basins) *B*_1_,...,*B_m_*. Since the attractors are subsets of the basins,

(9) $$\pi \left(A_{k}\right)=\pi \left(A_{k}\left|B_{k}\right.\right)\pi \left(B_{k}\right).$$

Therefore, we can compute the steady-state probability of any attractor *A_k_* by the following two steps: (1) the steady-state probability of basin *B_k_*, *π*(*B_k_*), and (2) the conditional probability of attractor *A_k_* given its being in *B_k_*, *π*(*A_k_*|*B_k_*).

##### (I). Obtaining the Steady-State Probability of Basin, *π*(*B_k_*)

Define a random variable *τ*(*t*) which measures the time elapsed between the last perturbation and the current time *t*. *τ*(*t*) = 0 means a perturbation occurs at *t*. For any starting state **h**, let

(10) $$P_{B_{i}}^{*}\left(B_{k}\right)=\lim_{t\to \infty }\mathrm{Pr}\left\{{\text{X}}_{t}\in B_{k}\left|{\text{X}}_{t-1}\in B_{i},{\text{X}}_{0}=\text{h},\tau \left(t\right)=0\right.\right\},$$

and define the conditional probability of being in state x∈B given that the system is inside a set *B*, prior to a perturbation,

(11) $$\pi^{*}\left(\text{x}\left|B\right.\right):=\lim_{t\to \infty }\mathrm{Pr}\left\{{\text{X}}_{t-1}=\text{x}\left|{\text{X}}_{t-1}\in B,{\text{X}}_{0}=\text{h},\tau \left(t\right)=0\right.\right\}.$$

The following theorem represents the steady-state distribution of the basins as the solution of a group of linear equations, where the coefficients are $P_{B_{i}}^{*}\left(B_{k}\right)$ 's. The lemma that follows gives the formula for the coefficients.

**Theorem 1**

(12) $$\pi \left(B_{k}\right)=\sum_{i=1}^{m}P_{B_{i}}^{*}\left(B_{k}\right)\pi \left(B_{i}\right).$$

**Lemma 1**

(13) $$P_{B_{i}}^{*}\left(B_{k}\right)=\sum_{\text{x}\in B_{k}}\sum_{\text{y}\in B_{i}}P_{\text{y}}^{*}\left(\text{x}\right)\pi^{*}\left(\text{y}\left|B_{i}\right.\right),$$

where $P_{\text{y}}^{*}\left(\text{x}\right)$ is the probability that state transition goes from **y** to **x** in one step by gene perturbation.

Now the only unknown is π^*^(**y**|*B_i_*). When *p* is small, the system spends majority of the time inside an attractor, and we can use the following approximation,

(14) $$\pi^{*}\left(\text{y}\left|B_{i}\right.\right)\approx \frac{1}{\left|A_{i}\right|}1_{\left[\text{y}\in A_{i}\right]},$$

where |*A_i_*| is the cardinality of *A_i_*. Therefore,

(15) $$P_{B_{i}}^{*}\left(B_{k}\right)\approx \frac{1}{\left|A_{i}\right|}\sum_{\text{x}\in B_{k}}\sum_{\text{y}\in A_{i}}P_{\text{y}}^{*}\left(\text{x}\right).$$

##### (II). Obtaining the Steady-State Probability of Attractor, *π*(*A_k_*)

**Lemma 2 ** For basin *B_k_*, initial state **h**, and fixed value *j*≥0,

(16) $$\lim_{t\to \infty }\mathrm{Pr}\left\{{\text{X}}_{t-j}=\text{x}\left|{\text{X}}_{t-j}\in B_{k},{\text{X}}_{0}=\text{h},\tau \left(t\right)=j\right.\right\}=\frac{1}{\pi \left(B_{k}\right)}\sum_{i=1}^{m}\sum_{\text{y}\in B_{i}}P_{\text{y}}^{*}\left(\text{x}\right)\pi^{*}\left(\text{y}\left|B_{i}\right.\right)\pi \left(B_{i}\right).$$

**Lemma 3 ** If *δ*(**x**,*A_k_*) is the number of iterations of **f** needed to reach the attractor *A_k_* from the state **x**, then for any $\text{x}\in A_{k},b<1$ ,

(17) $$\sum_{j=\delta \left(\text{x},A_{k}\right)}^{\infty }\left(1-b\right)b^{j}=b^{\delta \left(\text{x},A_{k}\right)}.$$

Applying the two lemmas and letting *b*=(1-*p*)*^n^*, we can obtain the steady-state probability of attractor *A_k_*.

**Theorem 2**

(18) $$\pi \left(A_{k}\right)=\sum_{i=1}^{m}\left[\sum_{\text{x}\in B_{k}}\sum_{\text{y}\in B_{i}}P_{\text{y}}^{*}\left(\text{x}\right)\pi^{*}\left(\text{y}\left|B_{i}\right.\right){\left(1-p\right)}^{n\delta \left(\text{x},A_{k}\right)}\right]\pi \left(B_{i}\right).$$

When *p* is small, using the approximation in Eq. (14), we have

(19) $$\pi \left(A_{k}\right)\approx \sum_{i=1}^{m}\frac{1}{\left|A_{i}\right|}\left[\sum_{x\in B_{k}}\sum_{y\in B_{i}}P_{y}^{*}\left(\text{x}\right){\left(1-p\right)}^{n\delta \left(x,A_{k}\right)}\right]\pi \left(B_{i}\right).$$

#### 3.2.2. Steady-State Distributions of Attractors in a PBN

In a PBN, we represent the pair (**x**,**f**) as the state of a homogeneous Markov chain, (**X***_t_*,**F***_t_*), and the transition probabilities are defined as

(20) $$P_{y,g}\left(\text{x},\text{f}\right)=\mathrm{Pr}\left\{{\text{X}}_{t+1}=\text{x},{\text{F}}_{t+1}=\text{f}\left|{\text{X}}_{t}=\text{y},{\text{F}}_{t}=\text{g}\right.\right\}$$

Assume the PBN is composed of *r* BNs *β*_1_(*V*,**f**_1_),...,*β_r_*(*V*,**f***_r_*). Within BN *β_k_*, the attractors and basins are denoted *A_ki_* and *B_ki_*, *i* = 1,...,*m_k_*. The computation of the steady-state probabilities are now split into three steps: (1) steady-state probabilities *π*(*B_ki_*,**f***_k_*) of the basins, (2) conditional probabilities *π*(*A_ki_*,**f***_k_*|*B_ki_*,**f**_k_), and (3) approximation to the marginal steady-state probabilities *π*(*A_ki_*) (since different BNs may have the same attractor).

The computations in steps (1) and (2) are similar to that of BNp, with (*B_ki_*,**f***_k_*) in place of *B_k_* whenever applicable, and there is one extra summation $\sum_{k=1}^{r}$ for the *r* component BNs. Interested readers can find details in [11].

From steps (1) and (2), we can obtain *π*(*A_ki_*,**f***_k_*). The last step sums up *π*(*A_ki_*,**f***_l_*) over *l* whenever the *l* -th BN has *A_ki_* as an attractor,

(21) $$\pi \left(A_{\mathrm{ki}}\right)=\sum_{l=1}^{r}\pi \left(A_{\mathrm{ki}},{\text{f}}_{l}\right).$$

Since *π*(*A_ki_*,**f***_l_*) is unknown when *k ≠ l*, we use the following approximation when *p* is small,

(22) $$\pi \left(A_{\mathrm{ki}},{\text{f}}_{l}\left|A_{\mathrm{lj}},{\text{f}}_{l}\right.\right)\approx \frac{\left|A_{\mathrm{ki}}\cap A_{\mathrm{lj}}\right|}{A_{\mathrm{lj}}}.$$

Thus,

(23) $$\pi \left(A_{\mathrm{ki}},{\text{f}}_{l}\right)\approx \sum_{j=1}^{m_{l}}\pi \left(A_{\mathrm{ki}},{\text{f}}_{l}\left|A_{\mathrm{lj}},{\text{f}}_{l}\right.\right)\pi \left(A_{\mathrm{lj}},{\text{f}}_{l}\right)\approx \sum_{j=1}^{m_{l}}\frac{\left|A_{\mathrm{ki}}\cap A_{\mathrm{lj}}\right|}{\left|A_{\mathrm{lj}}\right|}\pi \left(A_{\mathrm{lj}},{\text{f}}_{l}\right),$$

and

(24) $$\pi \left(A_{\mathrm{ki}}\right)\approx \sum_{l=1}^{r}\sum_{j=1}^{m_{l}}\frac{\left|A_{\mathrm{ki}}\cap A_{\mathrm{lj}}\right|}{\left|A_{\mathrm{lj}}\right|}\pi \left(A_{\mathrm{lj}},{\text{f}}_{l}\right).$$

### 3.3. Relationship Between PBNs and Bayesian Networks

Bayesian networks (BaN) are graphic models that describe the conditional probabilistic dependencies between variables, and have been used to model genetic regulatory networks [12]. An advantage of BaNs is that they involve model selection to optimally explain the observed data [2]; BaNs can use either continuous or discrete variables, which is more flexible for modeling. In comparison, Boolean models have explicit regulatory rules that carry biological information, which can be more appealing to biologist than the statistic representation of BaNs. Although Boolean models use binary-quantized variables which sets a limitation on the data usage, they are computational less complex than BaNs when learning the network structure from data (see Section 3.3 of [2] for a more detailed discussion and references). Since network structure learning is out of scope of this article, interested readers can refer to [12] for Bayesian learning, [13] for Boolean network learning, and [8, 14] for PBN learning.

While BNs are deterministic, PBNs and BaNs are related by their probabilistic nature; like PBNs, dynamic BaNs can be considered as Markov chains too. In the following analysis, we will show that equivalence between PBNs and BaNs can be established under certain conditions [15]. In this analysis, the random gene perturbation rate *p* in PBN is assumed to be 0.

A BaN with *n* random variables *X*_1_,...,*X_n_* (not necessarily binary) is represented by *Ba*(*H*,Θ), where ***H*** is a directed acyclic graph whose vertices correspond to the *n* variables and Θ is a set of conditional probability distributions induced by graph ***H***. Letting **X** = (*X*_1_,...,*X_n_*), *x_i_* be a realization of the random variable *X_i_*, and Pa(*X_i_*) be the parents of *X_i_*, the unique joint probability distribution over the *n* variables is given by

$\mathrm{Pr}\left\{x_{1},...,x_{n}\right\}=\prod_{i=1}^{n}\mathrm{Pr}\left\{x_{i}\left|\mathrm{Pa}\right.\left(X_{i}\right)\right\}.$

A dynamic Bayesian network (DBN) is a temporal extension of BaN, and consists of two parts: (1) an initial BaN *Ba*_0_ = (*H*_0_,Θ_0_) that defines the joint distribution of the variables *x*_1_(0),...,*x_n_*(0), and (2) a transition BaN *Ba*_1_ = (*H*_1_,Θ_1_) that defines the transition probabilities $\mathrm{Pr}\left\{\text{X}\left(t\right)\left|\text{X}\left(t-1\right)\right.\right\},\forall t$ . Let **x** represent a realization of **X**, and the joint distribution of **X**(0),...,**X**(*T*) can be expressed by

(25) $$\mathrm{Pr}\left\{\text{x}\left(0\right),...,\text{x}\left(T\right)\right\}=\mathrm{Pr}\left\{\text{x}\left(0\right)\right\}\prod_{t=1}^{T}\mathrm{Pr}\left\{\text{x}\left(t\right)\left|\text{x}\left(t-1\right)\right.\right\}=\prod_{i=1}^{n}\mathrm{Pr}\left\{x_{i}\left(0\right)\left|\mathrm{Pa}\left(X_{i}\left(0\right)\right)\right.\right\}\cdot \prod_{t=1}^{T}\prod_{j=1}^{n}\mathrm{Pr}\left\{x_{j}\left(t\right)\left|\mathrm{Pa}\left(X_{j}\left(t\right)\right)\right.\right\}.$$

In a PBN *G*(*V*,**F**,**c**), where $V=\left\{x_{1},...,x_{n}\right\},x_{i}\in \left\{0,1\right\}$ and **F** = {**f**_1_,...,**f**_*r*_}, the joint probability distribution of states over the time period [0,*T*] can be expressed as

$\mathrm{Pr}\left\{\text{x}\left(0\right),...,\text{x}\left(T\right)\right\}=\mathrm{Pr}\left\{\text{x}\left(0\right)\right\}\prod_{t=1}^{T}\mathrm{Pr}\left\{\text{x}\left(t-1\right)\to \text{x}\left(t\right)\right\}.$

For an independent PBN,

(26) $$\mathrm{Pr}\left\{\text{x}\left(0\right),...,\text{x}\left(N\right)\right\}=\mathrm{Pr}\left\{\text{x}\left(0\right)\right\}\prod_{t=1}^{T}\prod_{i=1}^{n}\mathrm{Pr}\left\{\text{x}\left(t-1\right)\to x_{i}\left(t\right)\right\}.$$

#### 3.3.1. An Independent PBN as a Binary-Valued DBN

Let the independent PBN be *G*(*V*,Ψ*α*) (the alternative representation, see what follows Definition 2). First, since a BaN can represent arbitrary joint distribution, the distribution of the initial state of PBN, Pr{**x**_0_} , can be represented by some *Ba*_0_. Second, to construct *Ba*_1_(*H*_1_,Θ_1_) from the PBN, we let set $X_{j}^{\left(i\right)}⫅V$ denote the regulators of gene *x_i_* in function $\psi_{j}^{\left(i\right)}$ ,

(27) $$\mathrm{Pa}\left(x_{i}\right)=\cup_{j=1}^{l\left(i\right)}{\text{X}}_{j}^{\left(i\right)}.$$

We construct graph *H*_1_ such that there are two layers of nodes, the first layer has nodes *X*_1_(*t*–1),...,*X_n_*(*t*–1), the second layer has nodes *X*_1_(*t*),...,*X_n_*(*t*), and there exists a directed edge from Xkt−1 to Xit if ∃j∈1,....,li in the PBN such that $x_{k}\in {\text{X}}_{j}^{\left(i\right)}$ . Thus in *H*_1_, Pa(*X_i_*(*t*)) corresponds to the set of all possible regulators of *x_i_* in the PBN.

Let *D_i_* be the joint distribution of the variables in Pa(*x_i_*), and recall that $\alpha_{j}^{\left(i\right)}=\mathrm{Pr}\left\{\psi_{j}^{\left(i\right)}\mathrm{issued}\right\}$ , then

(28) $$\mathrm{Pr}\left\{X_{i}=1\right\}=\sum_{j=1}^{l\left(i\right)}\mathrm{Pr}\left\{X_{i}=1\left|\psi_{j}^{\left(i\right)}\right.\text{is used}\right\}.\alpha_{j}^{\left(i\right)}$$

(29) $$=\sum_{j=1}^{l\left(i\right)}\left[\sum_{{\text{x}\in \left\{0,1\right\}}^{\left|\mathrm{Pa}\left(X_{i}\right)\right|}}D_{i}\left(\text{x}\right)\psi_{j}^{\left(i\right)}\left(\text{x}\right)\right].\alpha_{j}^{\left(i\right)}$$

(30) $$=\sum_{{\text{x}\in \left\{0,1\right\}}^{\left|\mathrm{Pa}\left(X_{i}\right)\right|}}D_{i}\left(\text{x}\right)\left[\sum_{j=1}^{l\left(i\right)}\psi_{j}^{\left(i\right)}\left(\text{x}\right)\alpha_{j}^{\left(i\right)}\right],$$

and we have

(31) $$\mathrm{Pr}\left\{X_{i}\left(t\right)=1\left|\mathrm{Pa}\left(X_{i}\left(t\right)\right)=\text{z}\right.\right\}=\sum_{j=1}^{l\left(i\right)}\psi_{j}^{\left(i\right)}\left(\text{z}\right)\alpha_{j}^{\left(i\right)}.$$

Eq. (31) defines Θ_1_ (induced by *H*_1_) for each node, thus any independent PBN *G*(*V*,Ψ*,α*) can be expressed as a binary DBN (*Ba*_0_,*Ba*_1_).

**Remark 2 **Strictly speaking, the input variables for $\psi_{j}^{\left(i\right)}$ are a subset of Pa(*x_i_*), so the notations in Eqs. (29-31) are not accurate when we use the same vector **x** (or **z**) for $\psi_{j}^{\left(i\right)}$ and for *D_i_* (or Pa(*X_i_*(*t*))). We should understand that those notations are only used as a convenience.

#### 3.3.2. A Binary-Valued DBN as an Independent PBN

Assume DBN (*Ba*_0_,*Ba*_1_) defined on **X**=(*X*_1_,...,*X_n_*) is given, and *X_i_*'s are binary-valued random variables. Now we demonstrate how to construct a PBN. Define the set of nodes *V* = {*x*_1_,...,*x_n_*} in PBN corresponding to *X*_1_,...,*X_n_*, and let the distribution of PBN initial state **x**_0_ = (*x*_1_(0),...,*x_n_*(0)) match Θ_0_ in *Ba*_0_(*H*_0_,Θ_0_).

In , *Ba*_1_(*H*_1_,Θ_1_), assume Pa(*X*_i_(*t*)) contains *k_i_* variables *X_i1_*,...,*X_ik_i__*. For each *X_i_*, we enumerate each conditional probability regarding *X_i_*(*t*) in Θ_1_ as a triplet zj,yj,pj, with zj∈0,1,yj=yj1yj2...yjki∈0,1ki,pj=PrXit=zjPaXit=yj and there are 2*^k^_i_*+1 such triplets. The triplets are arranged such that the first 2*^k_i_^* of them have *z_j_* = 1, and *p_j_*'s are in ascending order. For every *j*≤2*^k_i_^*, define a sequence of symbols $\tilde{{\text{x}}_{j}}=\tilde{x_{\mathrm{j1}}}\tilde{x_{\mathrm{j2}}}...\tilde{x_{{\mathrm{jk}}_{i}}}$ , where we choose the variable *x_jd_* for symbol $\tilde{x_{\mathrm{jd}}}\mathrm{if}y_{\mathrm{jd}}=1$ , and choose $\bar{x_{\mathrm{jd}}}$ (the negation of variable *x_jd_*) for symbol $\tilde{x_{\mathrm{jd}}}\mathrm{if}y_{\mathrm{jd}}=0$ .

Letting *l*(*i*)=2*^k_i_^*+1, we define the set of *l*(*i*) Boolean functions for gene *x_i_* in the PBN as $\psi_{i}=\left\{\psi_{1}^{\left(i\right)},...,\psi_{l\left(i\right)-1}^{\left(i\right)},\psi_{l\left(i\right)}^{\left(i\right)}\right\}$ , where

(32) $$\psi_{m}^{\left(i\right)}={\tilde{x}}_{m}\vee {\tilde{x}}_{m+1}\vee ...\vee {\tilde{x}}_{l\left(i\right)-1},\mathrm{for}1\leq m\leq l\left(i\right)-1$$

is a disjunction of conjunctions, and $\psi_{l\left(i\right)}^{\left(i\right)}$ is a zero function. Define the corresponding function selection probabilities α1i=p1,αmi=pm−pm−1for1<m≤li−1, and αlii=1−pli−1, it can be verified that

(33) $$\mathrm{Pr}\left\{X_{i}\left(t\right)=1\left|\mathrm{Pa}\left(X_{i}\left(t\right)\right)={\text{y}}_{j}\right.\right\}=\sum_{m=1}^{l\left(i\right)}\psi_{m}^{\left(i\right)}\left({\text{y}}_{j}\right)\alpha_{j}^{\left(i\right)}=\sum_{m=1}^{j}\psi_{m}^{\left(i\right)}\left({\text{y}}_{j}\right)\alpha_{j}^{\left(i\right)}=p_{1}+\sum_{m=2}^{j}\left(p_{m}-p_{m-1}\right)=p_{j}.$$

Therefore, a binary DBN can be represented as a PBN *G*(*V*,Ψ,*α*), where Ψ={Ψ_1_,...,Ψ*_n_*}, *α* = {*α*_1_,...,*α_n_*}, and $\alpha_{i}=\left(\alpha_{1}^{\left(i\right)},...,\alpha_{l\left(i\right)}^{\left(i\right)}\right)$ . It should be noted that the mapping from a binary DBN to an independent PBN is not unique, and the above representation is one solution.

Summarizing subsections 3.3.1 and 3.3.2, we have the following theorem [15].

**Theorem 3 **Independent PBNs *G*(*V*,Ψ,*α*) and binary-valued DBNs (*Ba*_0_,*Ba*_1_) whose initial and transition BNs *Ba*_0_ and *Ba*_1_ are assumed to have only within and between consecutive slice connections, respectively, can represent the same joint distribution over their common variables.

### 3.4. Structural Analysis

Boolean models, like any other networks, have two issues of interest: Is the model robust? Is the model controllable? From the standpoint of system stability, we require the model be robust, namely, resistent to small changes in the network; from the standpoint of network intervention, we desire that the network be controllable, such that it will respond to certain perturbation. There needs to be a balance of the two properties. These two questions encourage researchers to do the following, (1) Find structural properties of the network that are related to robustness and controllability; (2) Seek ways to analyze the effect of perturbations and to design control techniques.

In 3.4.1, (1) is addressed. We review some structural measures of Boolean models that quantify the propagation of expression level change from one gene to others (or vice versa). In 3.4.2, (2) is partly addressed, where we review structural perturbations, and present a methodology that analyzes the perturbation on Boolean functions. Since the control techniques are out of the scope of this paper, interested readers can find more information in the review articles [16, 17].

#### 3.4.1. Quantitative Measures of the Structure

In gene regulatory networks, the interactions among genes are reflected by two facts: the connections among genes, and the Boolean functions defined upon the connection. No matter it is the robustness or the controllability issue we are interested in, it all boils down to one central question: how a change in the expression level of one gene leads to changes in other genes in the network and vice versa. Here, we introduce three measures of the structural properties that are related to the question: canalization, influence and sensitivity.

When a gene is regulated by several parent genes through function *f*, some parent genes can be more important in determining its value than others. An extreme case is canalizing function, in which one variable (canalizing variable) can determine the function output regardless of other variables. 

**Definition 7 **[18] A Boolean function *f* : {0,1}*^n^* → {0,1} is said to be canalizing if there exists an $i\in \left\{1,...,n\right\}$ and $u,v\in \left\{0,1\right\}$ such that for all $x_{1},...,x_{n}\in \left\{0,1\right\}$ , if *x_i_* = *u* then *f*(*x*_1_,...,*x_n_*)=*v*.

In gene regulatory networks, canalizing variables are also referred to as the master genes. Canalization is commonly observed in real organisms, and it plays an important role in the stability of genetic regulation, as discussed in [19, 20]. Mathematically, researchers have shown that canalization is associated with the stability of Boolean networks. For more theoretical work, see [21-23].

Other than canalization, the degree of gene-gene interaction can be described in more general terms, and we define two quantitative measures, influence and sensitivity, as follows.

Consider a Boolean function *f* with input variables *x*_1_,...,*x_n_*. Letting **x** = (*x*_1_,...,*x_n_*), we define the influence of a gene on the function *f*.

**Definition 8** [2] The influence of a variable *x_j_* on the Boolean function *f* is the expectation of the partial derivative with respect to the distribution *D*(**x**),

(34) $$I_{j}\left(f\right)=E_{D}\left[\frac{\partial f\left(\text{x}\right)}{{\partial x}_{j}}\right]=\mathrm{Pr}\left\{\frac{\partial f\left(\text{x}\right)}{{\partial x}_{j}}=1\right\}=\mathrm{Pr}\left\{f\left(\text{x}\right)\neq f\left({\text{x}}^{\left(j\right)}\right)\right\}$$

Note that the partial derivative of *f* with respect to *x_i_* is

(35) $$\frac{\partial f\left(\text{x}\right)}{{\partial x}_{j}}=\left|f\left(\text{x}\right)-f{\left(\text{x}\right)}^{\left(j\right)}\right|,$$

in which **x***^(j)^* = (*x*_1_,...,1-*x_j_*,...,*x_n_*) (with *x_j_* toggled).

In a BN, since each node *x_i_* has one regulatory function *f_i_*, so the influence of node *x_j_* (assuming it regulates *x_i_*) on *x_i_* is *I_j_*(*x_i_*)=*I_j_*(*f_i_*). In a PBN, let the set of regulating functions for *x_i_* is $\psi_{1}^{\left(i\right)},...,\psi_{l\left(i\right)}^{\left(i\right)}$ , with function selection probabilities $\alpha_{1}^{\left(i\right)},...,\alpha_{l\left(i\right)}^{\left(i\right)}$ , the influence of gene *x_j_* on *x_i_*

(36) $$I_{j}\left(x_{i}\right)=\sum_{k=1}^{l\left(i\right)}I_{j}\left(\psi_{k}^{\left(i\right)}\right).\alpha_{k}^{\left(i\right)}.$$

Thus for a Boolean model with *n* genes, an *influence matrix* Γ of dimension *n* × *n* can be constructed, where its *i*,*j* element being Γ_*ij*_ = *I_i_*(*x_j_*). We can define influence of gene *x_i_* to be the collective influence of *x_i_* on all other genes,

(37) $$r\left(x_{i}\right)=\sum_{j=1}^{n}\Gamma_{\mathrm{ij}}.$$

Related to influence, we define the sensitivity of a function,

(38) $$s_{\text{x}}\left(f\right)=\sum_{j=1}^{n}\left|f\left(\text{x}\right)-f\left({\text{x}}^{j}\right)\right|.$$

Then the average sensitivity of *f* with respect to distribution *D* is

(39) $$s\left(f\right)=E_{D}\left[s_{\text{x}}\left(f\right)\right]=\sum_{j=1}^{n}E_{D}\left[\left|f\left(\text{x}\right)-f\left({\text{x}}^{\left(j\right)}\right)\right|\right]=\sum_{j=1}^{n}I_{j}\left(f\right).$$

The meaning of average sensitivity is that, on average, how much the function *f* changes between the Hamming distance one neighbors (i.e., the input vectors differ by one bit). For PBNs, the average sensitivity of gene *x_i_* is (cf. Eq. (37))

(40) $$s\left(x_{i}\right)=\sum_{j=1}^{n}I_{j}\left(x_{i}\right)=\sum_{j=1}^{n}\Gamma_{\mathrm{ji}}.$$

Biologically, the influence of a gene indicates its overall impact on other genes. A gene with high influence has the potential to regulate the system dynamics and its perturbation has significant downstream effect. The sensitivity of a gene measures its stability or autonomy. Low sensitivity means that other genes have little effect on it, and the “house-keeping” genes usually have this property [2]. It is shown that such quantitative measures (or variants) can help guide the control of genetic networks [24] and aid in the steady-state analysis [25].

#### 3.4.2. Structural Perturbation Analysis

There are two types of perturbation on Boolean models: perturbation on network states and perturbation on network structure. The former refers to a sudden (forced or spontaneous) change in the current state from **x** to **x**' , which causes the system dynamics to be disturbed temporarily. Such disturbance is transient in nature, because the network nodes and connections are intact, and the underlying gene regulation principles do not change. Therefore, the network attractors and the basins of attraction remain the same. However, if the perturbed Boolean model has multiple attractors, state perturbations may cause convergence to a different attractor than the original one, and may change the steady-state distribution of the network. This type of perturbation has been studied extensively (e.g. [26]), and finds its use in network control (e.g. [6]).

Perturbation on network structure refers to any change in the “wiring” or functions of the network. For instance, we may remove or add a gene to the network, change connections among genes, change the Boolean functions, or even change the synchronous Boolean network to an asynchronous model (where not all the genes are updated at the same time). Structural perturbation is more complex and less studied, compared to state perturbation. When network structure is perturbed, the network attractors and basins of attraction will be impacted, therefore the long-term consequence is more difficult to gauge than that of state perturbation.

The reasons for studying structural perturbation are: (1) modeling of gene regulatory networks is subject to uncertainty, and it is desirable to study the effect of small difference in network models on the network dynamic behavior; (2) it is likely that gene regulations, like other biological functions, have intrinsic stochasticity, and it is of interest to predict the consequence of any perturbation in regulation; (3) changing the network structure can alter the network steady-state distribution, thus structural perturbation can be an alternative way (with respect to state perturbation) of network control [25, 27, 28].

In [8], the authors developed theories to predict the impact of function perturbations on network dynamics and attractors, and main results are presented below. For more applications, see [28]. For further analysis in terms of steady-state distribution and application in network intervention, see [25].

**Problem formulation.** Given a Boolean network *β*(*V*,**f**), *V* = {*x*_1_,...,*x_n_*}, **f** = (*f*_1_,...,*f_n_*) , if one or more functions have one or more flips on their truth table outputs, we would like to predict the effect on state transitions and attractors.

Assume gene *x_i_* has *k_i_* regulators *x_i1_*,*x_i2_*,...,*x_ik_i__* then the truth table of *f_i_* has 2*^k_i_^* rows, as is shown below. The input vector on row *j* will be denoted ${\text{a}}_{j}^{i}\in {\left\{0,1\right\}}^{k_{i}}$ , for instance, ${\text{a}}_{1}^{i}=00...0$ . If we flip the output on row *j*, then we call it a one-bit function perturbation on *f_i_*, and denote it $f_{i}^{\left(j\right)}.$

**Table:** 

Row label	$x_{\mathrm{i1}}x_{\mathrm{i2}}\cdot \cdot \cdot x_{{\mathrm{ik}}_{i}}$	*f_i_*(.)
1	00⋅⋅⋅0	0
2	00⋅⋅⋅1	1
$\begin{array}{c} \cdot \\ \cdot \\ \cdot \end{array}$	$\begin{array}{c} \cdot \\ \cdot \\ \cdot \end{array}$	$\begin{array}{c} \cdot \\ \cdot \\ \cdot \end{array}$
$2^{k_{i}}$	11⋅⋅⋅1	0

Any state transition **s** → **w** contains *n* mappings, *f_i_* :**s** → *w_i_*. We define In*_i_*(s)=(*s_i1_*,*s_i2_*,...,*s_ik_i__*), which is a sub-vector of **s** that corresponds to the regulators of *x_i_*.

The following proposition and corollaries state the basic effects of one-bit function perturbation on the state transitions and attractors. Proofs and extensions to two-bit perturbations can be found in [28].

**Proposition 1 **A state transition **s** → **w** is affected by one-bit perturbation $f_{i}\to f_{i}^{\left(j\right)}$ if and only if ${\mathrm{In}}_{i}\left(s\right)={\text{a}}_{j}^{i}$ . If the state transition is affected, the new state transition will be **s** → **w**^(*i*)^, where **w**^(*i*)^ is defined to be the same as **w** except the *i* -th digit is flipped. 

**Corollary 1 **If *x_i_* has *k_i_* regulators, then the one-bit perturbation $f_{i}\to f_{i}^{\left(j\right)}$ will result in 2^*n-k_i_*^ changed state transitions in the state transition diagram. This is equivalent to 2^*n-k_i_*^ altered edges in the state transition diagram.

**Corollary 2 **(**Invariant singleton attractor**) Suppose state **S** is a singleton attractor. It will no longer be a singleton attractor following the one-bit perturbation $f_{i}\to f_{i}^{\left(j\right)}$ if and only if ${\mathrm{In}}_{i}\left(s\right)={\text{a}}_{j}^{i}$ .

**Corollary 3 **(**Emerging singleton attractor**) A non-singleton-attractor state **S** becomes a singleton attractor as a result of the one-bit perturbation $f_{i}\to f_{i}^{\left(j\right)}$ if and only if the following are true: (1) ${\mathrm{In}}_{i}\left(s\right)={\text{a}}_{j}^{i}$ , and (2) absent the perturbation, **S** → **S**^(*i*)^.

We use the following toy example to demonstrate the above results. From these results, more applications can be derived, such as controlling the network steady-state distribution through function perturbation, or identifying functional perturbation by observing phenotype changes [28].

**Example 1** Consider a BN with *n* = 3 genes,

(41) $$x_{1}\left(t+1\right)=x_{3}\left(t\right),$$

(42) $$x_{2}\left(t+1\right)=0,$$

(43) $$x_{3}\left(t+1\right)=\overset{-}{x_{1}}\left(t\right)x_{2}\left(t\right)+x_{1}\left(t\right)\overset{-}{x_{2}}\left(t\right),$$

where the truth table of *f*_3_ is shown below and the state transition diagram is shown in Fig. (**2**).

**Table:** 

Row label	*x*_1_	*x*_2_	*f*_3_(.)
1	0	0	0
2	0	1	1
3	1	0	1
4	1	1	0

If a one-bit perturbation forces *f*_3_ to become $f_{3}^{\left(3\right)}$ , since *k*_3_ = 2, 2 state transitions will be affected. By Proposition 1, states 100 and 101 no longer transit to 001 and 101 but to 000 and 100 respectively. Because of that, attractor cycle {001, 100} will be affected. Moreover, Corollary 2 predicts that the singleton attractor 000 is robust to the perturbation while 101 is not. The predictions are confirmed by the new state transition diagram shown in Fig. (**3**). 

Finally, the author would like to remind the readers that other works on (various types of) structural perturbation are available. For instance, in [29], the authors added a redundant node to Boolean network, such that the bolstered network is more resistent to a one-bit function perturbation (as defined above). In [30], the effect of asynchronous update of a *drosophila* segment polarity network model is examined in terms of the phenotypes (steady-states). In [25], the authors derived analytical results of how function perturbations affects network steady-state distributions and applied them to structural intervention. In [31], the author modeled gene knockdown and broken regulatory pathway in Boolean networks, and analyzed the effects.

## 4. SIMULATION ISSUES WITH BOOLEAN MODELS

Recall from Section 2 that a Boolean model of *n* genes has a finite state space, and a BN has deterministic dynamic behavior which can be fully captured by the state transition diagram. A PBN is probabilistic in nature, therefore its state transition is also probabilistic. For both BNs and PBNs, attractors are characteristic of their long-term behavior. Given the above knowledge, if we would like to know anything about a Boolean model, we should find out its state transition diagram and attractors first. This is to be discussed in Section 4.1.

For Boolean models, the most commonly encountered simulation issues include: (1) how to generate the time sequence data of a network, **x**(0),**x**(1),...,**x**(*t*),...; (2) how to find the network steady-state distribution if it exists; and (3) how to produce artificial Boolean models with prescribed attractors to facilitate other studies. Among them, (1) is a basic practice that can be utilized in (2) and (3), and we will deal with them in Sections 4.2, 4.3 and 4.4 respectively. Note that the techniques in 4.1 is crucial to all the three issues.

### 4.1. Generating State Transition Diagram and Finding Attractors

To obtain the state transition diagram of a BN, we first compile a state transition table. Assuming *n* nodes in the network, *x*_1_,...,*x_n_*, we evaluate the current state **x**(*t*) to be 00...0, 00...1, ..., 11...0, and 11...1 in turn, compute their respective **x**(*t*+1) 's, and tabulate the results. The states can also be represented by integers instead of binary vectors. Table **1** is an example when *n* = 3. In practice, we only store the second row (“next states”) for computational purpose, because by default the current states are always arranged such that they correspond to integers 0,1,2,...,2^*n*^–1.

To obtain the state transition diagram of a BN, we draw 2^*n*^ vertices, each representing a possible state, and connect two vertices by a directed edge if one state transits to the other based on the state transition table. If a state transits to itself, the edge points to itself. Fig. (**4**) is the state transition diagram based on Table **1**.

Similarly for a PBN, when gene perturbation rate *p*=0, we can draw its state transition diagram by combining the state transition diagrams of its component BNs. Now each edge has a probability attached to it, representing the possibility of one state transiting to the other. For example, if a PBN is composed of two BNs, where the first BN has state transitions shown in Table **1**, and the second BN has state transitions shown in Table **2**, and their selection probabilities are *c*_1_ and *c*_2_ = 1-*c*_1_ respectively, then when *p* = 0, the PBN's state transition diagram is shown in Fig. (**5**). When *p* > 0 , a state transition can either be driven by some network function or by random gene perturbations, and we may refer to its state transition matrix *T* when constructing the state transition diagram. It should be noted that the sum of probabilities of all the edges exiting a vertex should always be 1.

The following is a simple algorithm for finding the attractors of BN based on the state transition table (using integer representation of the states).

**Algorithm 1 **(Finding attractors)

1. Generate an array *a* of size 2^*n*^, and initialize all *a_i_*'S to 0. *a_i_* corresponds to state *i*–1.
2. Search for singleton attractors. For each state *i* between 0 and 2^*n*^–1, look up the (*i*+1) -th entry in the state transition table for its next state *j*. If *j* = 1, then *j* is a singleton attractor, set *a_j+1_* :=1.
3. Search for attractor cycles. For each state *i* between 0 to 2^*n*^-1, if *a_i+1_*=0, look up the state transition table repeatedly for the successor states of *i*, such that *i*→*j*→*k*... until a singleton attractor or an attractor cycle is reached. If an attractor cycle is reached, save the cycle states and set the corresponding elements in *a* to 1.

### 4.2. Simulating a Dynamic System

A common practice with a Boolean model defined on *V* = {*x*_1_,...,*x_n_*} is to generate time sequence data. **x**(0),**x**(1),**x**(2),.... A direct method is to start from an initial state **x**(0), and plug in the Boolean functions repeatedly to find the subsequent states (for BNs and PBNs), sometimes taking into consideration network switches and gene perturbations (for PBNs).

An alternative way, which is more efficient when simulation time is long $\left(t\gg 2^{n}\right)$ , is to utilize the information of state transition diagram (encoded in the state transition table) or transition probability matrix *T*. For BN, it entails converting the current state **x**(*t*) to an integer, and looking up the state transition table or matrix *T* for the next state **x**(*t*+1). For PBN, one can start from a randomly chosen initial state and a randomly chosen initial network (from *r* BNs), and follow either of the two protocols below. Note that we follow the notations in Definition 2 and use *p*, *q* to denote the random gene perturbation rate and network switching probability, respectively. Network selection probabilities are denoted by *c*_1_,...,*c_r_*.

- **Table-lookup and real-time computation based method.** Construct *r* state transition tables for the *r* component BNs respectively (letting gene perturbation rate *p* = 0). At any time *t*, if at the *k* -th network, generate *n* independent [0,1] uniformly distributed random numbers *p*_1_,...,*p_n_*. If *p_i_*<*p*, flip *x_i_*(*t*) to get $x_{i}\left(t+1\right);\mathrm{if}p_{i}>p\forall i$ (no gene perturbation), convert **x**(*t*) to integer and look up the *k* -th state transition table to find **x**(*t*+1). Finally, generate a [0,1] uniformly distributed random number *q_s_* and compare to *q* to decide if the system will switch network at *t*+1; if switch will occur, choose from the *r* networks according to the selection probabilities.
- *T*** matrix based method.** Compute the transition probability matrix *T*. If dec(**x**(*t*))=*i*–1, generate a [0,1] uniformly distributed random number *p_t_*. If $\sum_{l=1}^{j-1}t_{\mathrm{il}}\leq p_{t}<\sum_{l=1}^{j}t_{\mathrm{il}}$ (*t_il_* is the (*i,l*) element of *T*), then convert *j*–1 to a *n* -bit vector **s** (dec(**s**)=*j*–1) and the next state is **x**(*t*+1)=**S**.

One other issue of simulating a PBN is the choice of parameters *p* and *q*. As stated in Section 2, network switching probability *q* does not affect the probability of being at any constituent BN, and in theory we can choose any value for *q*; however, we prefer to choose small *q* because in a biological system, switching network corresponds to the change of context (reflecting a change of regulatory paradigm, either caused by environment change or internal signals), which should not occur very often. Moreover, if *q* is large, or even *q*=1, then network switching is frequent, and a short time sequence of data **x**(*t*_1_),**x**(*t*_1_+1),...,**x**(*t*_2_) are more likely to come from several BNs instead of from one single BN. This may pose a difficulty if we try to identify the underlying PBN and its component BNs from the sequence data [32]. On the other hand, if *q* is too small, and the number of BNs in the PBN is large, it will take too long a time to obtain the steady-state distribution by simulation method. Usually *p* should be small to reflect the rarity of random gene perturbation, and we let *p* <<*q*. Also, small *p* is helpful if the generated sequence data will be used as artificial time-series data for the identification the underlying PBN and its component BNs. However, if *p* is too small, it will take longer to obtain the steady-state distribution. Usually, we can choose *q* = 0.01 ~ 0.2 and *p* = 0.01% ~ 0.5%.

### 4.3. Obtaining the Steady-State Distributions

#### 4.3.1. Power Method

As discussed in Section 3.1, a PBN possesses a steady-state distribution when *q*, *p* > 0 [3]. By definition, this distribution π^*^ is the solution to linear equations *π* = *π*.*T* with constraint $\sum_{i}\pi_{i}=1$ is unique and can be estimated by iteration, given the transition probability matrix *T *(assuming *n* genes, and *N* = 2^*n*^ ).

**Algorithm 2 **(Finding steady-state distribution)

1. Set δ^*^ and generate an initial distribution $\pi^{\left(0\right)}=\left(\pi_{1}^{\left(0\right)},...,\pi_{N}^{\left(0\right)}\right)$ ; Let *k*:=0.
2. DO
  Compute *π*^(*k*+1)^:=*π*^(*k*)^.*T*;
  $\delta :=\left\|\pi^{\left(k+1\right)}-\pi^{\left(k\right)}\right\|;$
  *k*: = *k*+1;
  UNTIL (*δ*<δ^*^)
3. π^*^ :=*π*^(k)^.

Note that ||.|| can be any norm, such as ||.||_∞_.

When the number of BNs in a PBN is large and some BNs have small selection probabilities, an approximation method for constructing *T* is proposed in [33]. In the approximation, $\hat{T}$ is computed instead of *T*, which ignores *r*_0_ BNs whose selection probabilities *c_k1_*,...,*c_kr_0__* are less than a threshold value *ε*,

(44) $$\hat{T}=\overset{-}{T^{'}}+\tilde{T},$$

(45) $$\overset{-}{T^{'}}={\left(1-p\right)}^{n}.\sum_{j=1,j\neq k1,...,{\mathrm{kr}}_{0}}^{r}c_{j}T_{j}/\left(1-\sum_{i=1}^{r_{0}}c_{\mathrm{ki}}\right)$$

where *T_j_* (1≤ *j*≤ *r*) and $\tilde{T}$ are defined as in Eqs. (7) and (8). If $\hat{T}$ is used in place of *T*, and the solution for $\pi =\pi \hat{T}\text{ is }\hat{\pi^{*}}$ , the expected relative error in steady-state distribution is shown to be bounded by *O*(*ε*) [33]

(46) $$E\left[\frac{{\left\|\hat{\pi^{*}}-\hat{\pi^{*}}T\right\|}_{\infty }}{{\left\|\pi^{*}\right\|}_{\infty }}\right]<\left(2+2n\right)\sum_{i=1}^{r_{0}}c_{\mathrm{ki}}<2\left(n+1\right)r_{0}\varepsilon .$$

The following is an alternative method of obtaining steady-state distribution. If we are interested in the attractors only, knowing that the majority of the steady-state probability mass is on attractors if *p* is small, we may apply the Markov chain based analytic method in Section 3.2.

#### 4.3.2. Monte Carlo Simulation Method [34]

This method requires generation of a long time sequence of data, **x**(0),**x**(1),...,**x**(*T*), such that the frequencies of all the possible 2^*n*^ states approach the steady-state distribution. In a given *n* -gene PBN with gene perturbation rate *p*, the smaller *p* is and the larger *n* is, the longer it takes to converge to the steady-state distribution. In general, we need to simulate at least 10.2^*n*^.*p*^-1^ steps.

To estimate when the PBN has converged to its steady-state distribution, we can use the Kolmogorov-Smirnov test. The basic idea of Kolmogorov-Smirnov test is to measure the closeness of an empirical probability distribution to the theoretical distribution. Since the latter (steady-state distribution) is unknown in this case, we will test the closeness of two empirical distributions.

To get two quasi-i.i.d (independently and identically distributed) samples in PBN, we select two samples $\text{x}\left(t_{1}\right),\text{x}\left(t_{1}+m\Delta \right),...,\text{x}\left(t_{1}+\left(M-1\right)\Delta \right)\;\mathrm{and}\;\text{x}\left(t_{2}\right),\text{x}\left(t_{2}+m\Delta \right),...,x\left(t_{2}+\left(M-1\right)\Delta \right)\left(t_{1}<t_{2}\text{ and }t_{2}-t_{1}\neq m\Delta ,0<\forall m<M\right)$ , and the Kolmogorov-Smirnov statistic is defined as

(47) $$K=\frac{1}{M}\max_{\text{s}}\left|\sum_{m=0}^{M-1}1_{\left[00\cdot \cdot \cdot 0,\text{s}\right]\left(\text{x}\left(t_{1}+m\Delta \right)\right)-}\sum_{m=0}^{M-1}1_{\left[00\cdot \cdot \cdot 0,\text{s}\right]}\left(\text{x}\left(t_{2}+m\Delta \right)\right)\right|.$$

In the definition, the maximum is over the state space {0,1}^*n*^, and **1**_[00...0,s]_**(x)** is an indicator function whose output equals 1 if and only if $\text{x}\in \left\{00\cdot \cdot \cdot 0,\cdot \cdot \cdot ,\text{s}\right\},\text{s}\in {\left\{0,1\right\}}^{n}$ , and the output equals 0 otherwise.

### 4.4. Generating Artificial BNs with Prescribed Attractors [35]

In a simulation study of Boolean models, it is often necessary to create artificial networks with certain properties. Of special interest is the problem of generating artificial BNs with a given set of attractors, since attractors are hypothesized to correspond to cellular phenotypes and play an important role in the long term behavior of Boolean models.

First, note that the state transition diagram can be partitioned into level sets, where level set *l_j_* consists of all states that transit to one of the attractors in exactly *j* steps, and the attractors belong to the level set *l*_0_.

**Problem formulation** [35] Given a set of *n* nodes *V*={*x*_1_,...,*x_n_*}, a family of *n* subsets $P_{1},...,P_{n}\subseteq V$ with 0<*k*≤|*P_i_*|≤*K*, a set *A* of *d* states (binary vectors of *n* bits), and integers *l*,*L* satisfying 0<*l*<*L*, we will construct a BN defined on *V*, which satisfies the following constraints: the set of regulators of node *x_i_* is *P_i_* (*P*={*P*_1_,...,*P_n_*} is called the regulator set of *V*), the attractors are *A*_1_,...,*A_m_* such that $\cup_{j=1}^{m}A_{j}=A$ , and the BN has between *l* and *L* level sets.

Specifically, if we are interested in constructing a BN with only singleton attractors, its state transition diagram will be a *d* -forest (containing *d* single-rooted trees) if the BN has *d* singleton attractors. The following theorem gives the number of all possible state transition diagrams that only contain singleton attractors (the proof can be found in [35]).

**Theorem 4** The cardinality of the collection of all forests on *N* vertices is (*N*+1)^*N*-1^.

Since *N*=2^*n*^ and the number of all possible state transition diagrams are *N*^*N*^, when *n* is large, the ratio (*N*+1)^*N*-1^/*N*^*N*^ is asymptotically *e*/2^*n*^, thus a brute force search has a low success rate.

Assuming only singleton attractors are allowed, the following algorithm is for solving the search problem formulated above. A second algorithm is also given in [35], but shown to be less efficient.

**Algorithm 3 **(Generating artificial Boolean network)

1. Randomly generate or give in advance a set *A* of *d* states (as singleton attractors).
2. Randomly generate a predictor set *P*, where each *P_i_* has *k* to *K* nodes. If Step 2 has been repeated more than a pre-specified number of times, go back to Step 1.
3. Check if the attractor set *A* is compatible with *P*, i.e. only the attractors (each transits to itself) of the state transition diagram are checked for compatibility against *P*. If not compatible, go back to Step 2.
4. Fill in the entries of the truth tables that correspond to the attractors generated in Step 1. Using the predictor set *P* and randomly fill in the remaining entries of the truth table. If Step 4 has been repeated more than a pre-specified number of times go back to Step 2.
5. Search for cycles of any length in the state transition diagram *D* based on the truth table generated in Step 4. If a cycle is found go back to Step 4, otherwise continue to Step 6.
6. If *D* has less than *l* or more than *L* level sets go back to Step 4.
7. Save the generated BN and terminate the algorithm.

## 5. CLOSING WORDS

This paper has presented the following analysis and simulation issues of Boolean networks and probabilistic Boolean networks, which are models for gene regulatory networks.

- **Analysis**. An important aspect of Boolean models is that they can be viewed as homogeneous Markov chains; for a PBN, when the network switching probability *q* > 0 and gene perturbation rate *p* > 0, it possesses a steady-state distribution. Markov analysis serves as a basis for finding the steady-state probabilities of attractors and for proving the equivalence of PBN and dynamic Bayesian networks. Finally, a structural analysis is provided, where quantitative measures of gene-to-gene relationships are introduced, and the effect of perturbation on Boolean functions are analyzed.
- **Simulation**. Central to the simulation of Boolean models is the use of state transition diagram and transition probability matrix. In network simulation, different methods are presented and simple guidelines of parameter selection are provided. To test the convergence of a simulated PBN to its steady-state distribution, we can employ Kolmogorov-Smirnov statistic. Lastly, an algorithm for generating artificial BNs with prescribed attractors is presented.

To find more references on Boolean models, and obtain a MATLAB toolbox for BN/PBN, readers can go to the following website, http://personal.systemsbiology.net/ilya/PBN/PBN.htm. Another online source of papers is http://gsp.tamu.edu/Publications/journal-publications.

## Figures and Tables

**Fig. (1):**
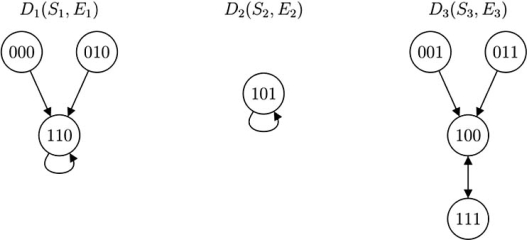
A state transition diagram *D*(*S, E*).

**Fig. (2):**
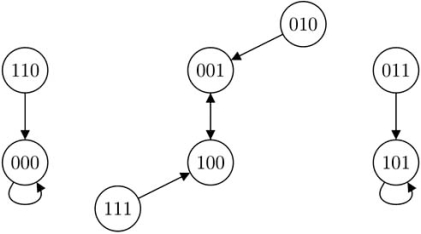
State transition diagram of the original BN, Example 1.

**Fig. (3):**
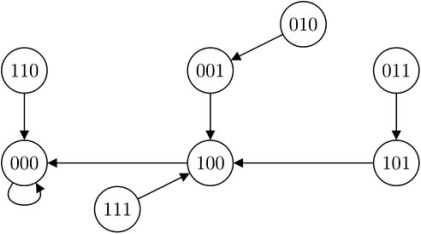
State transition diagram of the perturbed BN, Example 1.

**Fig. (4):**
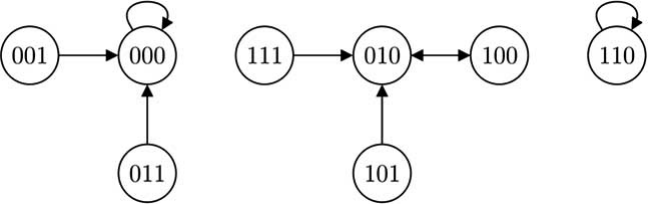
State transition diagram of a Boolean network.

**Fig. (5):**
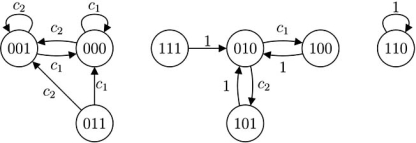
Example of state transition diagram of a probabilistic Boolean network when gene perturbation rate *p* = 0.

**Table 1:** Example of a State Transition Table for a BN

**Current states **	000	001	010	011	100	101	110	111
**Next states **	000	000	100	000	010	010	110	010

**Table 2:** State Transition Table for the Second BN in a PBN

**Current states**	000	001	010	011	100	101	110	111
**Next states**	001	001	101	001	010	010	110	010
